# Additional MRI for initial M-staging in pancreatic cancer: a cost-effectiveness analysis

**DOI:** 10.1007/s00330-021-08356-0

**Published:** 2021-11-27

**Authors:** Felix G. Gassert, Sebastian Ziegelmayer, Johanna Luitjens, Florian T. Gassert, Fabian Tollens, Johann Rink, Marcus R. Makowski, Johannes Rübenthaler, Matthias F. Froelich

**Affiliations:** 1 Department of Diagnostic and Interventional Radiology, Klinikum Rechts Der Isar, Technical University of Munich, Ismaninger Str. 22, 81675 München, Germany; 2 Department of Radiology, University Hospital, LMU Munich, Marchioninistr. 15, 81377 Munich, Germany; 3 Department of Radiology and Nuclear Medicine, University Medical Centre Mannheim, Theodor-Kutzer-Ufer 1-3, 68167 Mannheim, Germany

**Keywords:** Pancreatic neoplasms, Cost-effectiveness, Cancer staging, Magnetic resonance imaging, Multidetector computed tomography

## Abstract

**Objective:**

Pancreatic cancer is portrayed to become the second leading cause of cancer-related death within the next years. Potentially complicating surgical resection emphasizes the importance of an accurate TNM classification. In particular, the failure to detect features for non-resectability has profound consequences on patient outcomes and economic costs due to incorrect indication for resection. In the detection of liver metastases, contrast-enhanced MRI showed high sensitivity and specificity; however, the cost-effectiveness compared to the standard of care imaging remains unclear. The aim of this study was to analyze whether additional MRI of the liver is a cost-effective approach compared to routinely acquired contrast-enhanced computed tomography (CE-CT) in the initial staging of pancreatic cancer.

**Methods:**

A decision model based on Markov simulation was developed to estimate the quality-adjusted life-years (QALYs) and lifetime costs of the diagnostic modalities. Model input parameters were assessed based on evidence from recent literature. The willingness-to-pay (WTP) was set to $100,000/QALY. To evaluate model uncertainty, deterministic and probabilistic sensitivity analyses were performed.

**Results:**

In the base-case analysis, the model yielded a total cost of $185,597 and an effectiveness of 2.347 QALYs for CE-MR/CT and $187,601 and 2.337 QALYs for CE-CT respectively. With a net monetary benefit (NMB) of $49,133, CE-MR/CT is shown to be dominant over CE-CT with a NMB of $46,117. Deterministic and probabilistic survival analysis showed model robustness for varying input parameters.

**Conclusion:**

Based on our results, combined CE-MR/CT can be regarded as a cost-effective imaging strategy for the staging of pancreatic cancer.

**Key Points:**

• *Additional MRI of the liver for initial staging of pancreatic cancer results in lower total costs and higher effectiveness.*

• *The economic model showed high robustness for varying input parameters.*

## Introduction

Pancreatic cancer is an exceptionally aggressive tumor entity with the lowest 5-year survival rate of all solid tumors [1]. In addition to pronounced heterogeneity, poor prognosis can be attributed primarily to delayed diagnosis, such that 50% of pancreatic cancers are already metastatic at initial diagnosis [2]. Due to the increasing incidence, it is predicted that pancreatic cancer will become the second leading cause of cancer-related death in the USA by 2030, therefore posing a relevant burden to the healthcare systems [3]. Surgical resection usually in terms of pancreaticoduodenectomy followed by adjuvant chemotherapy is the only curative therapeutic option. Despite steady improvements in surgical technique and perioperative management, resection of pancreatic cancer remains a demanding procedure with a postoperative mortality rate of 3–5% [4, 5]. In the metastatic stage, patients receive palliative chemotherapy with gemcitabine/nab-paclitaxel or FOLFIRINOX. In particular, FOLFIRINOX was shown to significantly prolong survival, however, at the expense of increased toxicity [6, 7]. The potential adverse effects of the therapeutic options emphasize the relevance of correct TNM classification, especially with regard to the presence of metastasis, which is a contraindication for surgical resection. For M-staging, the liver as the most frequent site of metastasis is of particular importance [8]. Staging of pancreatic cancer involves biphasic computed tomography of the chest, abdomen, and pelvis to evaluate resectability and rule out metastasis. The detection rate of liver metastases in computed tomography is described in the literature with a sensitivity of 70 to 76% [9, 10]. Contrast-enhanced MRI is frequently described as an alternative for assessing the locoregional extent and detecting lymph node and liver metastases. It appears to be dominant over contrast-enhanced computed tomography (CE-CT) in detecting liver lesions, with a sensitivity of 90 to 97% [11, 12]. Furthermore, additional MR imaging during the staging of pancreatic cancer was shown to reduce resection rates, indicating that patients in a metastatic stage who received staging with CE-CT were resected incorrectly [13].

In this context, additional imaging is often deemed expensive. A cost-effectiveness analysis is a tool to assess the impact of potential changes in patient management and its impact on long-term costs and effectiveness. Despite improvement in diagnosis and treatment of pancreatic cancer, no study has been performed to evaluate the utilization of combined contrast-enhanced MRI and CT compared to standard imaging (contrast-enhanced CT) in the detection of features for non-resectability from an economic point of view. Therefore, the aim of our study was to determine the cost-effectiveness of combined CE-MRT/CT in detecting liver metastasis at the initial staging of pancreatic cancer compared to the standard of care imaging (SCI) using CE-CT.

## Methods

### Model structure

A decision model based on Markov simulations was developed using dedicated analysis software (TreeAge Pro Version 19.1.1) to evaluate the cost-effectiveness of each imaging strategy. For the simulation, the Markov model included the following states:

- Alive, non-resectable: i.e., describing patients with initially metastatic disease or non-resectable primary tumor, therefore not undergoing surgery
- Alive, resected, no metastasis or local recurrence: i.e., describing patients after resection without the presence of metastasis or local recurrence
- Alive, resected, presence of R1-situation or local recurrence: i.e., describing patients without metastasis but local recurrence or R1-situation after resection
- Alive, resected, presence of metastasis (with and without local recurrence): i.e., describing patients with presence of metastasis, either associated with recurrence or due to missed metastatic disease on pre-surgical imaging
- Dead

### Input parameters

Ethics approval was not necessary for this retrospective analysis based on commonly available data. The model input parameters were estimated based on evidence from published literature. Age-specific risk of death was derived from the US life tables [14]. The probability to correctly classify tumors as resectable using CE-CT was set to 92.25%; consequently, the false positive rate was 7.75% [13]. The cost of pancreatic resection with respect to potential complications and differing patient characteristics was set to $42,869 [15], which poses a reasonable estimate between $22,000 stated by Sutton et. al and $55,538–$61,806 by Tramontano et. al [16, 17]. All input parameters and corresponding references are listed in Table 1.

**Table 1:** Model input parameters

Variable	Estimate	Source
Expected age at diagnostic procedure	70 years	[18]
Assumed willingness-to-pay per QALY	$100,000	Assumption
Discount rate	3%	Assumption
Markov model time horizon	5 years	Assumption
Diagnostic test performances		
CT probability of TP	92.25%	[13]
CT probability of FP	7.75%	[13]
Costs (acute)		
CT chest, abdomen, pelvis	$692	Medicare (Ref.No.: 71260 + 74,177)
MRI abdomen	$615	Medicare (Ref.No.: 74183) [19]
Surgery	$42,869	[15]
Costs (long term)		
Therapy for patients with M1	$60,000	[17, 20]
Therapy/follow-up after surgery	$36,126 (first year); $1,126 (following years)	Adapted from [17]
Therapy after resection with M1	$60,000	[17, 20]
Therapy with local recurrence / R1	$30,000	[17]
Utilities		
M1 after surgery	0.6	[21, 22]
M1 without surgery	0.65	[23]
M0 post surgery	0.79 (first year), 0.87 (following years)	Adapted from [21, 23]
Death	0	Assumption
Transition probabilities		
Proportion of R1-resections	80%	[24]
Occurrence of metastasis after resection	38.00%	[25]
Mortality rate of surgery	3.70%	[4]
Mortality rate with M1 cancer	50.74%	[18]
Probability of death M0 cancer	2.90%	[14]

The Willingness-to-pay (WTP) was set to $100,000 per quality-adjusted life year (QALY) at a discount rate of 3% [26, 27].

### Costs and utilities

Starting from the United States (US) healthcare perspective, diagnostic procedure costs were estimated based on Medicare data and available literature (Table 1). Annual costs for patients with respect to different therapy regimens and tumor states were derived from recent literature [17, 20].

Utility was measured in the quality-adjusted life years (QALY) in follow-up after each diagnostic strategy. According to previous studies, quality of life (QOL) for resected patients without metastasis was set to 0.726 in the first year and 0.797 for the following years [21, 22]. For patients with metastasis and with or without surgery, QOL was set to 0.65 and 0.6 respectively [23].

### Transition probabilities

Transition probabilities were derived from a systematic review of the recent literature (Table 1). The perioperative mortality rate within 90 days of pancreatic resection was set to 3.7% [4]. The probability of secondary occurrence of metastases after resection of the primary tumor was estimated to be 38% per year [25]. The annual mortality rate of patients with and without metastasis was set to 50.74% and 2.9% respectively [14, 18].

### Cost-effectiveness analysis

The cost-effectiveness analysis was simulated for Markov run time of 5 years after the initial staging of pancreatic cancer. QALY and costs were calculated for the base case scenario with respect to WTP and discount rate.

To evaluate model uncertainty, deterministic and probabilistic sensitivity analysis was conducted. The deterministic sensitivity analysis was performed by altering the input parameters and observing their influence on the incremental effectiveness, incremental cost, and incremental cost-effectiveness ratio (ICER).

The Monte Carlo modeling was used for probabilistic sensitivity analysis. A total of 30,000 iterations were performed to estimate acceptability curves. Furthermore, the net monetary benefit (NMB) with respect to the probability of possible tumor resection was calculated for both imaging strategies.

## Results

### Cost-effectiveness analysis

**Fig. 1:**
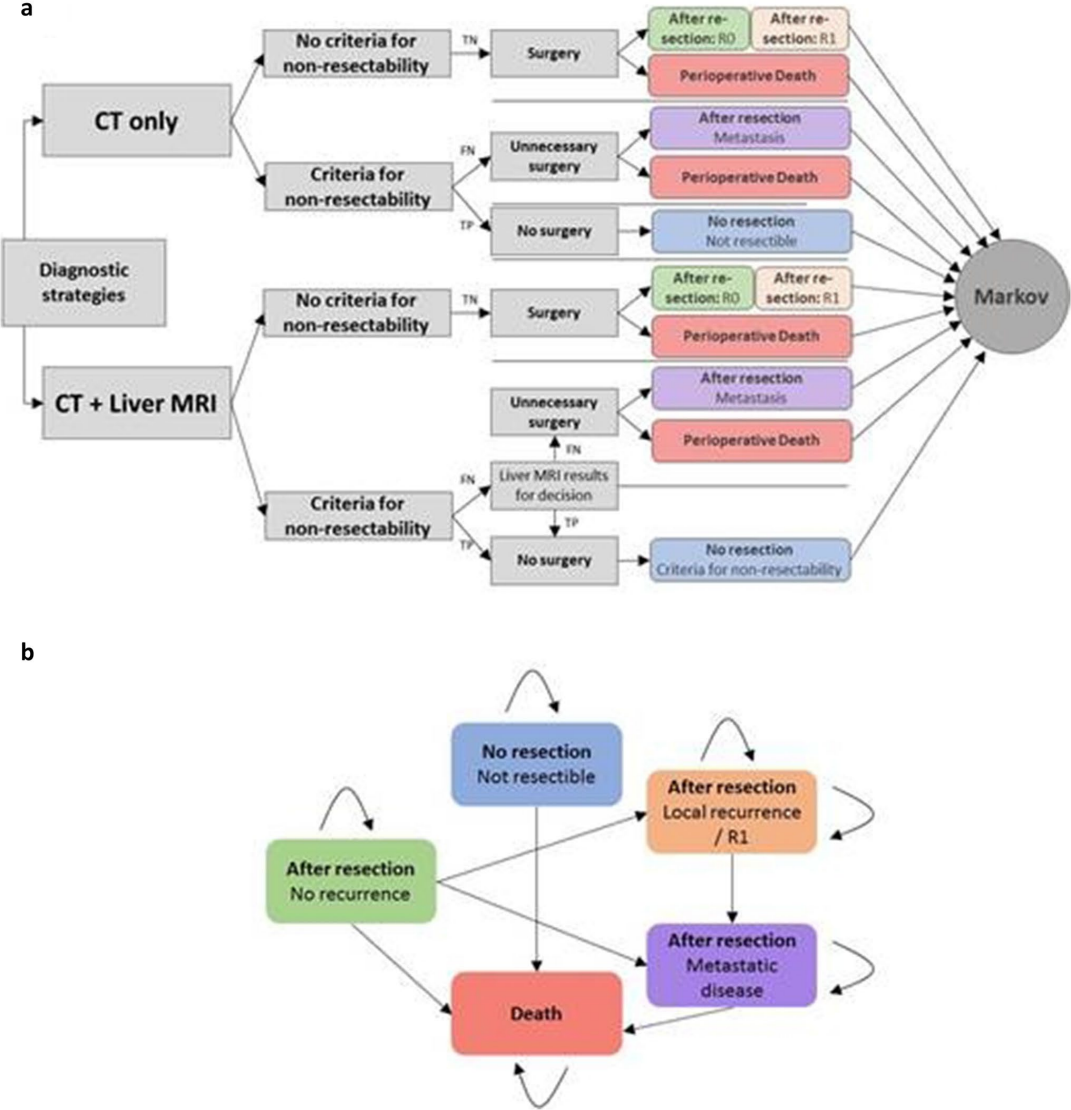
**a** Effective alternative to CE-CT schematic overview of the decision model for both diagnostic strategies (CE-CT and CE-MR/CT). Markov model analysis was conducted for each outcome. **b** The Markov model with the respective states and their potential transition. The initial state was determined by the outcome in the decision model. TP, true positive; TN, true negative; FP, false positive; FN, false negative, CT, computed tomography; MRI, magnetic resonance imaging

The decision model and the respective schematic architecture of the Markov model with the potential states of disease are shown in Fig. 1. For the base case scenario with a WTP of $ 100,000 per QALY and a 5-year time span, the model yielded a total cost and effectiveness of $187,601 and 2.337 QALYs for the SCI (CE-CT), whereas combined CE-MR/CT was estimated to cost $185,597 with an effectiveness of 2.347 QALYs. The calculated Incremental cost-effectiveness ratio for combined CE-MR/CT was negative, indicating higher effectiveness of CE-MRI/CT at lower costs, i.e., the dominance of this strategy over the alternative. The NMB for CE-MR/CT was $49,133 and $46,117 for CE-CT.

### Probabilistic sensitivity analysis

**Fig. 2:**
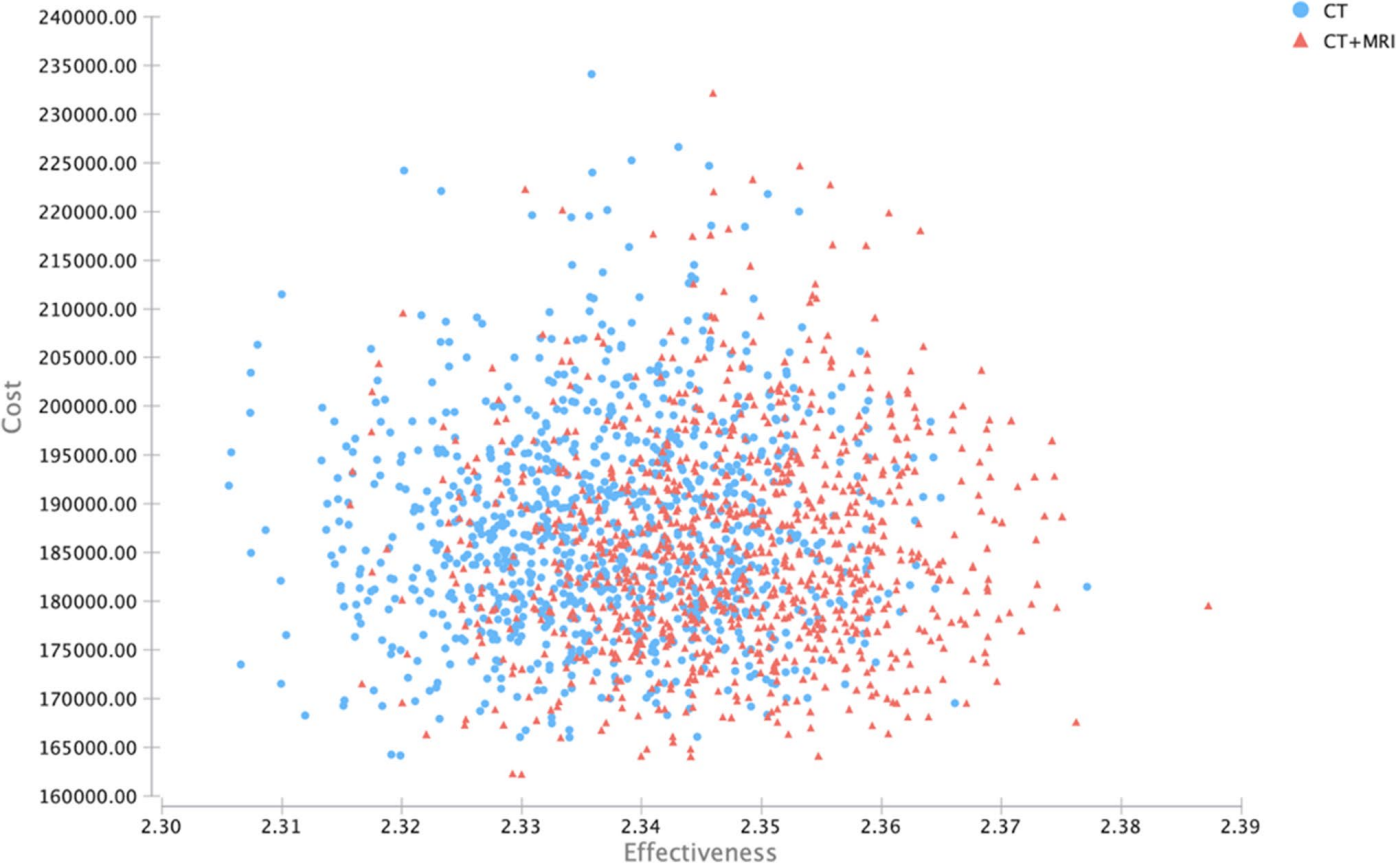
Scatterplot of cost and effectiveness of CE-MR/CT and CE-CT for exemplary iterations. CT, computed tomography; MRI, magnetic resonance imaging

At the WTP threshold of $100,000 per QALY combined CE-MR/CT remained the cost-effective alternative to CE-CT in the majority of all iterations. Even for a hypothetical reduction in WTP thresholds to $0, combined CE-MR/CT remained the cost-effective alternative in the vaster majority of iterations. Exemplary iterations of the model with the corresponding costs and effectiveness of the respective modalities are shown in Fig. 2.

### Deterministic sensitivity analysis

**Fig. 3:**
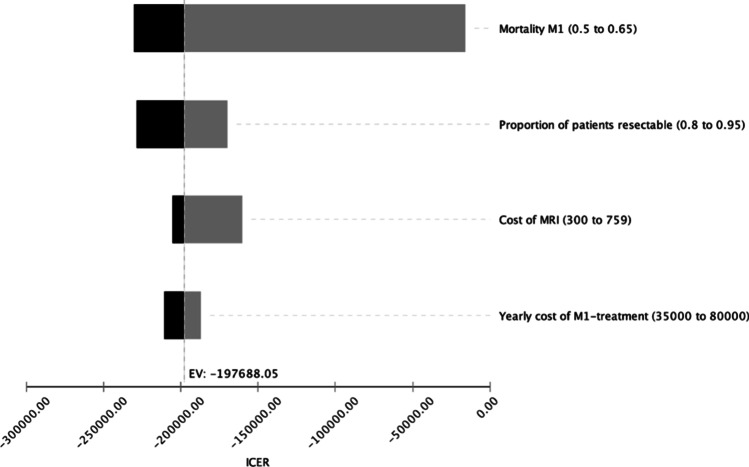
Results of the deterministic sensitivity analysis visualized as a tornado diagram, showing the influence of input parameter variation on the incremental cost-effectiveness ratio (ICER). MRI, magnetic resonance imaging; EV, expected value at base case scenario; M1, metastasized

**Fig. 4:**
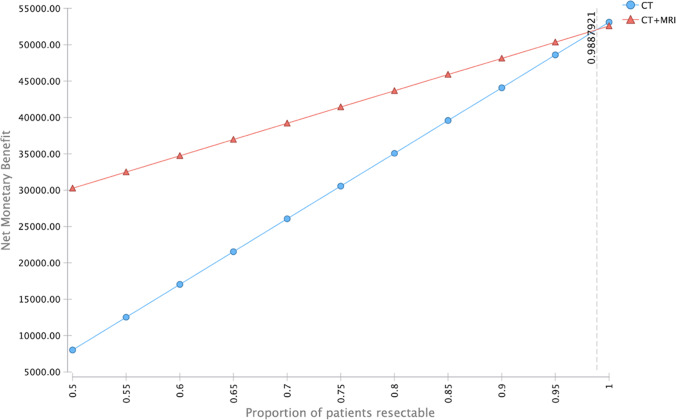
Sensitivity analysis of the net monetary benefit (NMB) with respect to the probability of possible tumor resection. CE-MR/CT has a higher NMB up to a hypothetical resectability rate of higher than 0.98

A deterministic sensitivity analysis was performed to account for possible variance in induced costs as well as different probabilities of state transition. The incremental cost-effectiveness ratio remained below the WTP threshold for the applied changes in the above-mentioned parameters, indicating the cost-effectiveness of combined CE-MR/CT in the detection of features for non-resectability for the most common variants of the respective parameters. A tornado diagram displaying the changes in ICER is shown for each parameter in Fig. 3. A dedicated one-way sensitivity analysis was performed, to investigate the influence of the proportion of patients resectable. For the broad majority of inputs, i.e., levels below 98.88%, CT + MRI yielded the higher net monetary benefit when compared to CT alone (Fig. 4).

## Discussion

Due to the increasing incidence combined with extensive and cost-intensive treatment options, the economic burden of pancreatic cancer on the healthcare system is steadily increasing. The detection of liver metastases is of particular importance as they are the most frequent metastases and an exclusion criterion for surgical resection and its associated costs and complications. In the present study, we show that CE-MR/CT is a cost-effective diagnostic strategy in staging pancreatic cancer. The superiority of MRI compared to CT in the diagnosis and staging of pancreatic cancer has already been demonstrated [28, 29]. However, despite the fact that cost-effectiveness is of increasing importance in health care, studies evaluating the economic implications of various imaging modalities in pancreatic cancer remain sparse. Pamela et al. were able to show that CE-CT, followed by laparoscopy and laparoscopic ultrasonography, was a cost-effective diagnostic workup in determining resectability of pancreatic cancer with MRI imaging coming second [30]. However, this analysis was based on lower sensitivity for MRI, potentially due to the state of scanner development at that time. Diffusion-weighted imaging and hepatocyte-specific contrast agent were able to greatly increase the sensitivity of MRI in liver lesion detection [31, 32]. Heinrich et al. postulated that ^18^F FDG PET-CT is the cost-effective method over CE-CT for assessing the resectability of pancreatic cancer [33]. Whether this method is also dominant over CE-MRI was not evaluated. In our study, we did not integrate ^18^F FDG PET-CT as an additional imaging strategy due to the higher sensitivity and specificity at lower cost of CE-MRI reported in the literature. Furthermore, the cost-effectiveness of CE-MR in the detection of liver metastases in comparison to different imaging modalities has already been demonstrated for other tumor entities [34].

In our study, combined CE-MR/CT was the dominant strategy compared to CE-CT for a range of WTP-thresholds. Consequently, it would be the dominant diagnostic workup even for lower WTP-thresholds, as in the UK health care system, for instance [35].

Deterministic sensitivity analysis showed a consistent negative ICER for CE-MR/CT for different variations in the input parameters, indicating lower costs at higher effectiveness. In addition, robustness is emphasized by a positive NMB for CE-MR/CT under varying resection probabilities. Despite promising results, the following limitations of our study have to be addressed. It must be emphasized that cost-effectiveness analysis with decision-based models is highly dependent on the input parameters used, and thus an optimal decision for each individual case is not achievable due to deviating parameters. The Markov model used did not differentiate between tumor extent, as this would exceed the scope of the study. Due to the correlation between tumor stage and present metastases, the cost-effectiveness of imaging modalities with respect to tumor stage should be investigated. Moreover, our decision model did not show an extra group for patients with undetected metastases in MRI in the Markov model as input values would be unlikely to be available in the literature. False-negative diagnosis in MRI therefore is reflected through the rate of recurrence after resection of these patients. Functional imaging modalities as ^18^F FDG PET-CT or PET-MRI were excluded from the study due to the lack of establishment in clinical practice. In the following studies, these modalities should be investigated in more detail based on promising results from recent findings [36, 37]. Lastly, this study was performed based on guidelines for the implementation of cost-effectiveness analyses and therefore analyses costs and effectiveness for an average of patients. As a matter of course, not every individual patient’s history can be taken into account, and treatment decisions should always be based on individual considerations.

In our study, we show that combined CE-MR/CT is a cost-effective strategy for the staging of pancreatic cancer as compared to SCI using CE-CT. This finding was robust even considering realistic variations in induced costs as well as different probabilities of state transition.

## Abbreviations

CE-CT: Contrast-enhanced computed tomography
ICER: Incremental cost-effectiveness ratio
NMB: Net monetary benefit
QALY: Quality-adjusted life year
QOL: Quality of life
SCI: Standard of care imaging
WTP: Willingness-to-pay

## Declarations

### Guarantor

The scientific guarantor of this publication is PD Dr. med. Matthias Frölich.

### Statistics and biometry

Matthias Frölich has significant statistical expertise regarding cost-effectiveness analysis.

### Methodology

• retrospective

• observational

• multicenter study
